# Hallmarks of Sublethal Endothelial Injury Are Differentially Induced by *Cuminum cyminum* Extracts with Distinct Phytochemical Profiles

**DOI:** 10.3390/cimb48030255

**Published:** 2026-02-26

**Authors:** Margarita L. Martinez-Fierro, Virginia Flores-Morales, Idalia Garza-Veloz

**Affiliations:** Doctorado en Ciencias con Orientación en Medicina Molecular, Unidad Académica de Medicina Humana y C.S., Campus UAZ Siglo XXI, Universidad Autónoma de Zacatecas, Carretera Zacatecas—Guadalajara Km 6, Ejido La Escondida, Zacatecas 98160, Mexico; margaritamf@uaz.edu.mx

**Keywords:** sublethal endothelial injury, *Cuminum cyminum*, endothelial toxicity, phytochemical extracts

## Abstract

*Cuminum cyminum* (cumin) is widely used as a culinary spice and medicinal plant, yet its endothelial effects remain poorly defined, and viability-only toxicity tests may miss relevant changes. We evaluated whether four *C. cyminum* extracts (aqueous, methanolic, acetonic, hexane) induce sublethal endothelial injury (SEI), defined as preserved viability with functional, molecular, and morphological alterations. Human microvascular endothelial cells (HMEC-1) were exposed to increasing extract concentrations, and endothelial viability, migration, molecular responses, and cytomorphology were evaluated. Organic extracts (methanolic, acetonic, and hexane) induced endothelial stress and injury-related responses. Methanolic and acetonic extracts caused concentration- and time-dependent cytotoxicity, accompanied by reduced migration, stress-related gene modulation, and marked morphological damage, whereas the hexane extract induced pronounced cytomorphological disruption and strong *NOS2* induction. In contrast, the aqueous extract exhibited minimal cytotoxicity and significantly enhanced endothelial migration, together with *FGF2* upregulation and moderate *NOS2* induction, consistent with a sublethal, pro-migratory phenotype. Overall, extract composition strongly influenced endothelial responses, demonstrating that SEI can occur in the absence of overt cytotoxicity and highlighting the need to incorporate functional endothelial endpoints into the safety evaluation of herbal and plant-derived extracts.

## 1. Introduction

Medicinal plants and culinary spices are widely consumed worldwide due to their perceived health benefits, bioactive properties, and long-standing use in traditional medicine. *Cuminum cyminum* L. (Family Apiaceae) known as cumin, is a native plant to northern Egypt, the Mediterranean, Iran, and India; it is one of the most cultivated popular spices globally. Cumin is a small annual herb with slender, angular branched stem, it is a mixture of united and separated mericarps; yellowish green or yellowish brown, elongated ovoid [1]. This plant has been commonly used in traditional medicine to treat a variety of diseases because of its medicinal properties, which include antimicrobial activity, hypoglycemic, anti-inflammatory, cardioprotective, chemo-preventive, neuroprotective, and digestive stimulant action [2,3,4,5], largely attributed to its rich phytochemical composition, including terpenoids, phenolic compounds, flavonoids, and aldehydes [4,5,6,7,8,9,10]. Based on these properties, *Cuminum cyminum* (*C. cyminum*) has been traditionally employed for the management of a wide range of conditions, including gastrointestinal disorders (e.g., diarrhea, dyspepsia, and carminative use), inflammatory and respiratory conditions (e.g., cough, hoarseness, and general inflammation), metabolic and hepatic alterations (e.g., jaundice), urinary tract-related conditions (e.g., kidney and bladder stones, diuretic use), dermatological and wound-related conditions (e.g., ulcers and boils), as well as neurological and sensory complaints (e.g., headache and eye diseases) [10,11,12,13]. Despite its extensive dietary and ethnopharmacological use, the safety profile of *C. cyminum*, particularly at higher concentrations or in non-aqueous extracts, remains incompletely characterized.

Endothelial cells are regulators of vascular physiology, mediating vascular homeostasis, angiogenesis, inflammation, hemostasis, and redox balance through active biochemical signaling and dynamic responses to biomechanical cues in the vascular microenvironment [14,15]. They produce vasoactive mediators such as nitric oxide (NO) and prostacyclin to maintain vascular tone, regulate permeability, and limit thrombosis, thereby contributing to oxidative balance and proper blood flow [16]. Endothelial cells also coordinate leukocyte adhesion and transmigration during immune responses, integrating inflammatory signals with vascular function [16].

Endothelial dysfunction, defined as a shift toward reduced NO bioavailability, enhanced pro-inflammatory and pro-thrombotic phenotypes, and impaired vasodilatory capacity, is a critical early event in the pathogenesis of cardiovascular diseases such as atherosclerosis and hypertension [17]. Endothelial dysfunction is also implicated in chronic inflammatory disorders and has been associated with disease progression and complications in metabolic and vascular diseases [15]. Moreover, dysfunctional endothelium contributes to the tumorigenic process, where altered endothelial signaling can promote vascular inflammation, increased permeability, and a microenvironment conducive to tumor growth and metastasis [18]. Endothelial dysfunction can also favor pro-thrombotic states that are linked to cancer progression and resistance to therapy [19].

Given the central role of the endothelium in vascular homeostasis, the evaluation of plant-derived compounds requires approaches that extend beyond conventional cytotoxicity assays. Endothelial cells can undergo functional, molecular, and structural changes in response to bioactive exposures without overt loss of viability. When toxicity assessment relies exclusively on viability-based endpoints, these sublethal yet biologically relevant alterations may be overlooked, resulting in an underestimation of vascular risk.

While numerous investigations have focused on the beneficial biological activities of *C. cyminum* extracts, including antioxidant and anti-inflammatory properties, these studies have largely emphasized protective or therapeutic effects rather than potential endothelial toxicity or safety-related outcomes, and systematic evaluations on their direct cytotoxic effects on endothelial cells are limited and often inconsistent (frequently focusing on cancer cells or non-vascular models rather than endothelial systems) [20]. Therefore, a more thorough and standardized evaluation that considers extraction methods, solvent effects, and endothelial-specific models is necessary to clarify both the therapeutic potential and possible vascular toxicological risks of *C. cyminum* components. The present study aimed to evaluate, in vitro, the endothelial cytotoxic effects of four different *C. cyminum* extracts (aqueous, methanolic, acetonic, and hexane), integrating phytochemical profiling, cytotoxicity assays, wound healing assays, gene expression analysis, and in silico modeling. Rather than focusing on antioxidant or anti-inflammatory activity, this work specifically examines endothelial functional and molecular responses relevant to vascular integrity and safety assessment. We also introduced the concept of sublethal endothelial injury (SEI), defined as a biological state in which endothelial cell viability is preserved despite the presence of functional, molecular, and/or morphological alterations that precede overt cytotoxicity. This context enables discrimination between adaptive or stress-related endothelial activation and irreversible endothelial damage induced by plant-derived extracts. Although the phytochemical composition of *C. cyminum* extracts has been characterized in previous studies using advanced analytical techniques [9,21,22,23,24,25,26,27,28,29,30,31,32,33,34,35,36,37,38,39,40], the present work focuses on the endothelial biological responses elicited by extracts with distinct phytochemical profiles rather than on compound-level chemical characterization. This approach allows the evaluation of vascular-relevant effects and avoids duplication of previously reported analytical studies.

## 2. Materials and Methods

### 2.1. Preparation of Plant Extracts

Cumin plants (525 g of seeds) purchased from a quality seed store were transferred to the laboratory, sieved to remove impurity, washed with distilled water and dried in the shade for 8 days. The dried plant material was ground using a new, dedicated blender to avoid contamination from previously processed materials, and the resulting powder was passed through a clean, residue-free stainless-steel 250 µm sieve.

The extracts were prepared by sequential maceration using solvents of increasing polarity, specifically hexane, acetone, and ethanol, in order to progressively remove non-polar, moderately polar, and more polar phytochemical components. Solvents were grade ACS and purchased from J. T. Baker^®^ (Ciudad de Mexico, Mexico). Sequential maceration was selected to reduce overlap between phytochemical fractions and to facilitate polarity-based separation of extractable compounds. The times of each maceration were 24, 48 and 72 h.

Between each maceration step, the plant material was allowed to dry to complete solvent evaporation before exposure to the next solvent, thereby minimizing the risk of residual solvent carryover and cross-contamination between extracts. Subsequently, each extract was filtered using Whatman^®^ No. 10 filter paper (Cytiva, Maidstone, UK) and the solvent was removed by using a rotary evaporator (Büchi R-220, Ciudad de Mexico, Mexico).

Lyophilization was performed sequentially from extracts obtained with lower to higher polarity solvents to further reduce the possibility of cross-extract contamination. Each extract was then lyophilized for subsequent analysis of antimicrobial activity, cytotoxic effects, and endothelial cell migration.

### 2.2. Preliminary Phytochemical Analysis of C. cymium Extracts

The phytochemical screening performed in this study was intended solely to confirm the presence of major classes of secondary metabolites and does not represent a comprehensive chemical characterization of the extracts. Compound-level identification and quantification of *C. cyminum* constituents have been previously reported [9,21,22,23,24,25,26,27,28,29,30,31,32,33,34,35,36,37,38,39,40] and were not duplicated in the present study.

According to above, and with the aim to identify the main groups of metabolites present in the hexane, acetonic, methanolic and aqueous extracts of *C. cyminum*, an analysis by thin layer chromatography (TLC, Merk^®^ silica gel 60 F254 plates, Ciudad de Mexico, Mexico) was performed, using a series of chromogenic agents as specific chemical developers that allowed us to qualitatively identify the main families of secondary metabolites (SM), as well as functional groups present in the extracts. Among the SM and functional groups are flavonoids, alcohols, alkaloids, carbonyl groups, esters, and sugars, among others. Table 1 shows the chemical developers used and the way they reveal. The procedure described by Dominguez was used for its preparation [41].

For the preliminary phytochemical analysis (PPA), a 10 mg sample of the extracts was taken and dissolved in 0.5 mL of the corresponding solvent. Subsequently, tests were carried out with various solvent mixtures of different degrees of polarity, until the mixture was found with which the compounds were separated adequately and allowed good visualization. In this case, the most suitable mixture was Hex: EtOAc in proportions of 95:05, 80:20 and 50:50. Moreover, 156 Merck brand silica gel 60 F_254_ chromatographic plates of 1 cm wide by 4 cm long were used. The dry plates were impregnated with one of the various chromogenic agents using a cotton swab and for visualization they were placed on a heating rack at 100 °C, leaving on it the time necessary for the reaction to occur that allowed the stains to be visualized, or following the time established for each of the developers [41].

### 2.3. Cell Culture and Treatment Conditions

Human microvascular endothelial cells (HMEC-1) were cultured with Gibco™ DMEM (Dulbecco’s modified Eagle’s medium) medium, 10% fetal bovine serum, 1% Anti-Anti Gibco™ (10,000 µg/mL streptomycin, 25 µg/mL amphotericin B and 10,000 units/mL penicillin), 10 mM glutamine, 1 µg/mL hydrocortisone and 10 ng/mL EGF (endothelial growth factor) based on ATCC^®^ guidelines. Cells were cultured in a humidified environment with 5% CO_2_ at 37 °C in Thermofisher^®^ incubator (ThermoFisher Scientific, Waltham, MA, USA). After reaching 90% confluence in the 75 cm^2^ culture flask, 0.8% ethylenediaminetetraacetic acid-trypsin was used to extract the cells, for 3 min at 37 °C and seeded on plates of the corresponding assays. Cell counting was performed in a Neubauer chamber with trypan blue staining and the total number of cells was obtained by multiplying the average number of cells by the dilution factor. Preparation of MTT (3-(4,5-Dimethylthiazol-2-yl)-2,5-Diphenyltetrazolium Bromide) Invitrogen^®^ reagent (Thermo scientific, Waltham MA, USA) at a concentration of 12 mM or 5 mg/mL, 25 mg of MTT powder was weighed and dissolved in 5 mL of sterile 1X PBS, then the solution was filtered and stored at 4 °C. The aqueous extract was diluted in sterile 1X PBS, the methanolic and acetonic extract was diluted in 100% DMSO and, all the solutions were filtered with a 0.2 µm filter and storage at 4° C.

### 2.4. Cell Viability Assay

Cell viability was evaluated by MTT assay. MTT method is a non-radioactive colorimetric assay to measure cell viability, cytotoxicity or proliferation. Metabolically active cells can convert the MTT in a yellow water-soluble tetrazolium die to a water-insoluble dark purple formazan by reductive cleavage of the tetrazolium ring [42]. The cell viability assay was carried out as follows: HMEC-1 cells were seeded in a 96-well plate with a total of 1 × 10^4^ in each well with 100 µL of culture medium. The following concentrations were used for all aqueous, methanolic, and acetonic extracts: 0.2 µg/µL, 0.4 µg/µL, 0.8 µg/µL, 1.6 µg/µL, 3.2 µg/µL, and 6.4 µg/µL. This concentration screening was designed to capture the full spectrum of biological responses, from preserved viability to cytotoxicity. In each assay, untreated cells were included as controls, and PBS 1X alone, or with 0.05% DMSO. The extracts were added to each corresponding well and incubated for 24 and 48 h. For the MTT assay, 10 µL per well were added and incubated in a humidified environment with 5% CO_2_ at 37 °C for 4 h in the absence of light. Afterwards, the supernatant was removed from each well and 50 µL of 100% DMSO was added to dissolve the formed crystals. The absorbance was measured at 570 nm using a spectrophotometer plate reader (SmartReader 96; Accuris Instruments). All experiments were performed in triplicate.

### 2.5. Determination of IC_50_ Values

The half maximal inhibitory concentration (IC_50_) was calculated to quantify the concentration-dependent cytotoxic effects of *C. cyminum* extracts on HMEC-1 cells. IC_50_ values were estimated from concentration–response curves generated using cell viability data obtained at 24 and 48 h of exposure. Calculations were performed using the online Quest Graph™ LD50 Calculator (AAT Bioquest Inc., Sunnyvale, CA, USA) [43], adapted to determine inhibitory concentrations rather than lethal doses. The IC_50_ was defined as the concentration required to reduce cell viability by 50% relative to the untreated control. IC_50_ values were determined only for extracts that produced sufficient inhibitory responses within the tested concentration range, while IC_50_ estimation was not feasible for extracts lacking measurable inhibition.

### 2.6. Gene Expression Assay

HMEC-1 cells were seeded in a 96-well plate and cultured to 80% confluence, yielding approximately 2.6 × 10^4^ cells/well. Individual wells of cells, in triplicate, were exposed to aqueous, methanolic, acetonic, and hexane extracts at concentrations of 0.8, 1.6, 3.2, and 6.4 µg/µL. Untreated cells, receiving only culture medium, served as controls. Cells were incubated at 37 °C, 5% CO_2_ for 48 h. Total RNA was isolated from each triplicate group according to the RNeasy Mini Kit protocol (QIAGEN, Hilden, Germany). cDNA was synthesized from total RNA using High-Capacity cDNA Reverse Transcription Kit and random hexamers. The RNA concentration was measured using a NanoDrop 2000 spectrophotometer (Thermo Fisher Scientific, Wilmington, DE, USA). Quantitative real-time PCR (qRT-PCR) was performed using a StepOnePlus Real-Time PCR Systems in 96-well PCR plates. Fifty nanograms of synthesized cDNA were used as templates for qRT-PCR amplification in a 20 μL final reaction volume using SYBR^®^ Select Master Mix (all reagents and instruments were from ThermoFisher Scientific, Waltham, MA, USA), and 500 nM gene-specific primers (T4 Oligo, Irapuato, Mexico), which were designed based on the respective GenBank sequence for the examined gene. Amplification was performed under the following thermal cycling conditions: predenaturation for 10 min at 95 °C, PCR amplification for 40 cycles of denaturizing for 15 s at 95 °C, and annealing for 1 min at 60 °C. Cycle series were followed by melt-curve analyses to check the specificity of the reaction. Sequences and product sizes of forward and reverse primers for *BCL2* associated x apoptosis regulator (*BAX*), fibroblast growth factor 2 (*FGF2*), nitric oxide synthase 2 (*NOS2*), cyclin-dependent kinase inhibitor 1A (*P21*), tumor protein P53 (*P53*), vascular endothelial growth factor (*VEGFA*), superoxide dismutase 1 (*SOD1*), and glyceraldehyde-3-phosphate dehydrogenase (*GAPDH*) are listed in Supplementary Table S1. Primer efficiency and specificity were confirmed through standard curve analysis and melting profile evaluation, with amplification efficiency assessed relative to *GAPDH*; all this accords with a standardization reported before [44].

### 2.7. Endothelial Cell Migration Assay

HMEC-1 cell migration was measured with the wound assay, where cells were seeded in 24-well plates with a total of 2.5 × 10^5^ cells per well with 500 µL of culture medium; for the experimental groups the concentrations 1.6 µg/µL and 3.2 µg/µL of the aqueous, methanolic, acetonic and hexane extracts were used. These concentrations were selected considering their IC_50_ and because the methanolic, acetonic extracts showed a decrement on cell viability compared to the cells without treatment. For the cell culture assays, when the aqueous extract was tested, only culture medium was added to the cells without treatment, whereas that, for the ethanolic, acetonic, or hexane extracts, the control cells used as reference were added with culture medium plus 1% DMSO. Cell cultures were incubated in a humidified environment with 5% CO_2_ at 37 °C overnight. After having adherence and cell confluence, they were treated with mitomycin at a concentration of 4 ng/mL for 1 h and incubated at 37 °C. Wounds were made at the bottom of the wells by using a 100 µL tip. Afterwards, the supernatant was discarded and replaced by fresh medium with the different extracts and incubated. All the treatments were evaluated in duplicate. Photographs were taken with an inverted fluorescence microscope at 0, 1, and 24 h of exposure to the extracts; the area of the wound was measured with ImageJ™ software v1.54d (National Institutes of Health, Bethesda, MD, USA), some points forming a square marked on the bottom of the plate on the outside were used as a reference.

### 2.8. Cytological and Morphological Evaluation of HMEC-1 Cells

To assess cytological and morphological changes induced by exposure to *C. cyminum* extracts, HMEC-1 cells were seeded on chamber slides and cultured to approximately 50% confluence. Cells were exposed to aqueous, methanolic, acetonic, or hexane extracts at a final concentration of 0.8 µg/µL and incubated for 24 h at 37 °C under standard culture conditions. Following exposure, cellular morphology was initially evaluated by direct observation under light microscopy and images captured with an Axiocam 503 color camera, attached to an Axio Observer Z1 motorized inverted fluorescence microscope (All Carl Zeiss, Jena, Germany). Cells were then fixed and stained using the Papanicolaou staining method according to the manufacturer’s instructions. Stained slides were examined using a Carl Zeiss microscope (Axio Observer Z1), and cytological features were assessed, including cell shape, cell–cell contact, cytoplasmic integrity, nuclear size and morphology, chromatin condensation, and the presence of morphological features associated with cell damage or death, such as nuclear pyknosis, karyorrhexis, and cytoplasmic fragmentation.

### 2.9. In Silico Modeling of Components of Cuminum cyminum

#### 2.9.1. Phytochemical Group Enrichment Analysis

To compare the dominant phytochemical profiles associated with each extraction strategy, a literature-based phytochemical group enrichment analysis was performed using compositional data reported for *C. cyminum* aqueous, methanolic, acetonic, and hexane extracts. Compounds identified in each extract were grouped into major phytochemical classes (phenolic acids, flavonoids, tannins/saponins, aromatic aldehydes, monoterpenes, fatty acids/esters, phenylpropanoids, and sesquiterpenes/triterpenes), based on their chemical structure and polarity. For each extract, the relative enrichment of each phytochemical group was categorized using a semi-quantitative, evidence-based scoring system reflecting both the consistency of compound reporting across independent studies and the availability of quantitative abundance data. Enrichment levels were assigned as follows: 0, not reported or insufficient evidence; 1, repeatedly identified but without individual quantitative values; and 2, compounds reported as major constituents or with quantitative abundance data. This scoring approach enabled standardized comparison of phytochemical group enrichment across extraction methods while avoiding direct comparison of heterogeneous quantitative metrics. The resulting scoring matrix was assembled using Microsoft Excel (Microsoft Corp., Redmond, WA, USA), and the comparative heatmap was generated using Python (v3.10) with the Matplotlib library (v3.8.2), allowing consistent visualization of phytochemical group enrichment across extraction strategies.

#### 2.9.2. In Silico Prediction of Biological Activity of Selected *C. cyminum* Compounds

The in silico analysis was conducted as a hypothesis-supporting approach based exclusively on phytochemical constituents of *C. cyminum* previously identified and validated in the literature. This approach does not imply experimental confirmation of compound presence in the tested extracts but rather provides a mechanistic approach to contextualize the observed biological effects.

The prediction of the biological activity for each selected compound was conducted using Pass Online tool. The Pass Online server (https://www.way2drug.com/passonline/pe.php#s4; accessed on 3 December 2025) is a network tool that predicts simultaneously 3678 kinds of activity with a mean accuracy of prediction of about 95% (leave-one-out cross validation) on the basis of the compound’s structural formula. The approach used in PASS (Prediction of Activity Spectra for Substances) is based on the suggestion that Activity = f(Structure). The PASS training set consists of over 260,000 drug-like biologically active compounds. They include information about drugs, drug-candidates, lead compounds and toxic compounds. Biological activity is the result of a chemical compound’s interaction with biological entity, using the corresponding SMILES. The interpretation of prediction results is if Pa > 0.7, the substance is very likely to exhibit the predicted activity in experiment. If 0.5 < Pa < 0.7, the substance is likely to exhibit the activity predicted in experiment, but the probability is less, and the compound is unlike known pharmaceutical agents. Finally, if Pa < 0.5, the compound is unlikely to exhibit the predicted activity [45,46].

### 2.10. Statistical Analysis

Data were described as mean ± standard deviation. Each extract’s areas of inhibition were compared using one-way analysis of variance (ANOVA) coupled to the Holm–Sidak method as a multiple comparison procedure, considering the selected antibiotic as a reference. Data obtained from the cell viability assay and the wound assay were analyzed with Shapiro–Wilk’s normality test; a one-way ANOVA with Tukey’s post hoc was performed to see if there were statistically significant differences. *p*-values less than 0.05 were considered significant. Statistical analysis was carried out in SigmaPlot software v12 (Systat Software Inc., San Jose, CA, USA).

## 3. Results

### 3.1. Percentage Yield of C. cyminum Extracts and Preliminary Phytochemical Analysis

A total of 20.4046 g, (4.08%), 19.4546 g (3.9%), 28.70 g (5.75%) and 16.155 g (3.23%) of hexane, acetonic, methanolic, and aqueous extracts of *C. cyminum* were obtained after freeze-drying procedures.

After obtaining extract and as an initial step in the identification of bioactive chemical features, a PPA was performed. This approach was selected because it allows rapid and specific detection of major phytochemical classes using simple, low-cost, and widely accessible assays, facilitating comparative analysis among extracts obtained with different solvents. Although PPA does not yield definitive identification of individual compounds, it was used because it provides valuable information regarding the major classes of phytochemicals present in the plant extracts, particularly in extensively studied species such as *C. cyminum*. The results of the phytochemical screening are summarized in Table 2.

Positive results were observed for the presence of alcohols, carbonyl compounds (aldehydes and ketones), alkaloids and derivatives, aldehydes and double bonds, amino acids, and quinones. In the case of alkaloids, of all four chromogenic agents used, a positive result was found in three of them.

The tests with 2,4-dinitrophenyl hydrazone and potassium permanganate were also positive, which indicates the presence of carbonyl groups, particularly of aldehydes and ketones. As mentioned above, the results of these tests are not entirely conclusive and a negative result does not definitively prove the nonexistence of the metabolites it identifies, since the results can be affected by the low sensitivity of the test to characterize small concentrations of a substance or by the possible superposition of some compounds on others, thus generating interference and preventing the chromogenic agent from reacting with its corresponding compound.

### 3.2. Assessment of Cell Viability

#### 3.2.1. Effects of *C. cyminum* Extracts on HMEC-1 Cell Viability

HMEC-1 cells were exposed to 0.2, 0.4. 0.6, 0.8, 1.6, 3.2 and 6.4 µg/µL of each *C. cymium* extract and the cell viability was evaluated at 24 and 48 h. It should be noted that these values correspond to crude plant extracts rather than purified compounds. As such, higher nominal concentrations are often required to elicit measurable biological responses in vitro. In this study the wide concentration range included allowed construction of complete dose–response curves for each extract, capturing transitions from preserved metabolic activity to cytotoxicity and enabling calculation of IC_50_ values under standardized conditions.

**Figure 1:**
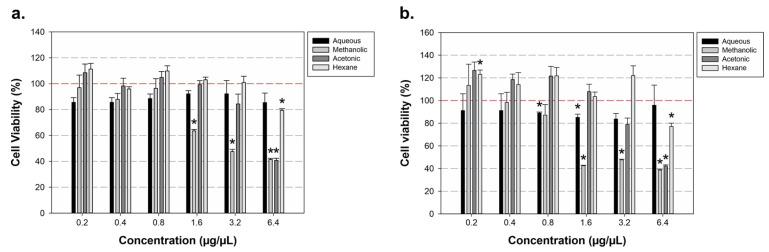
Evaluation of *Cuminum cyminum* extracts on HMEC-1 cell viability. (**a**) The 24 h results. Compared with the untreated control, exposure to the methanolic extract resulted in a significant reduction in cell viability at concentrations between 1.6 and 6.4 µg/µL. The acetonic extract also induced a significant decrease in viability, but only at the highest concentration tested (6.4 µg/µL). (**b**) The 48 h results. Relative to the untreated control, treatment with the aqueous extract led to a reduction in cell viability at concentrations ranging from 0.8 to 3.2 µg/µL. The methanolic extract maintained a concentration-dependent decrease in viability between 1.6 and 6.4 µg/µL, while the acetonic extract reduced viability at concentrations from 3.2 to 6.4 µg/µL. In contrast, the hexane extract produced a significant decrease in cell viability only at 6.4 µg/µL. Values above 100% may be a reflection of increased metabolic activity relative to control as measured by the MTT assay. * Significance indicated at *p* < 0.05. The red dashed line indicates 100% of cell viability.

The results of the cell viability assays were as follows: the aqueous extract ranged from 85.6% to 92.1% at 24 h and from 83.7% to 95.9% at 48 h (Figure 1a,b). For the methanolic extract, cell viability was observed between 41.4% and 96.9% at 24 h and between 37.2% and 109.2% at 48 h.

As shown in Figure 1, the acetonic extract showed cell viabilities from 40.8% to 108.4% at 24 h and from 40.7% to 121.9% at 48 h. Finally, the hexane extract had cell viability ranging from 79.4% to 111.2% at 24 h and from 74.3% to 118.5% at 48 h. At 24 h of exposure, a significant reduction in endothelial cell viability was observed following treatment with the methanolic extract at concentrations of 1.6, 3.2, and 6.4 µg/µL compared with the untreated control (*p* = 0.009, *p* = 0.002, and *p* = 0.001, respectively). In contrast, the acetonic extract induced a significant decrease in cell viability only at the highest concentration tested (6.4 µg/µL; *p* = 0.001). No statistically significant effects were observed for the aqueous or hexane extracts at this time point (Figure 1a).

After 48 h of exposure, a broader cytotoxic profile was evident. Treatment with the aqueous extract resulted in a significant reduction in cell viability at concentrations ranging from 0.8 to 3.2 µg/µL (*p* < 0.05). The methanolic extract continued to exert a pronounced cytotoxic effect, significantly decreasing cell viability across all tested concentrations (1.6–6.4 µg/µL; *p* = 0.001 for each concentration). Similarly, the acetonic extract significantly reduced cell viability at concentrations of 3.2 and 6.4 µg/µL (*p* < 0.05). Notably, the hexane extract also produced a significant decrease in endothelial cell viability, but only at the highest concentration evaluated (6.4 µg/µL; *p* = 0.005) (Figure 1b).

#### 3.2.2. Time-Dependent Effects of *Cuminum cyminum* Extracts on Endothelial Cell Viability

**Figure 2:**
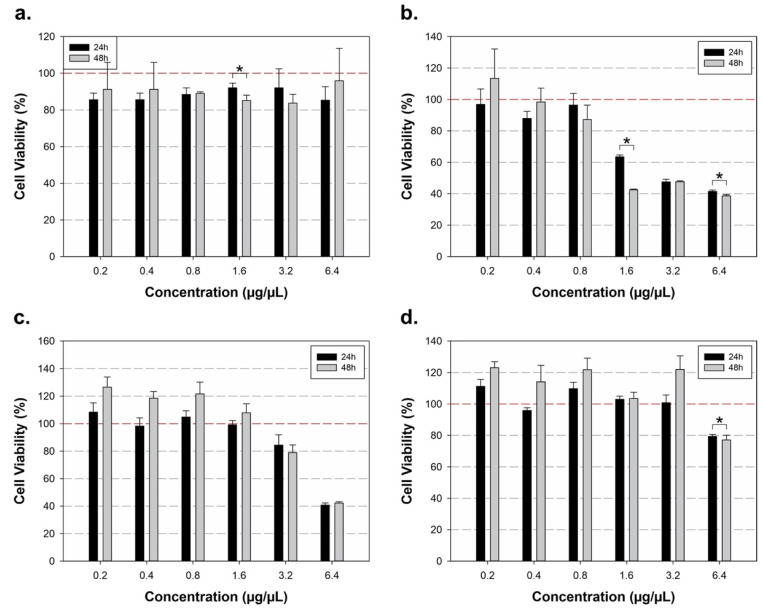
Impact of *Cuminum cyminum* extracts on HMEC-1 cell viability over time. This figure compares the effects of *C. cyminum* extracts on HMEC-1 cell viability following 24 h and 48 h of exposure, highlighting statistically significant differences between time points. (**a**) Aqueous extract: a significant decrease in cell viability was observed at 1.6 µg/µL (*p* = 0.013). (**b**) Methanolic extract: significant differences in cell viability were detected at concentrations of 1.6 and 6.4 µg/µL (*p* < 0.05). (**c**) Acetonic extract: no statistically significant changes in cell viability were observed across the evaluated concentrations between the two time points. (**d**) Hexane extract: a significant reduction in cell viability was detected at 6.4 µg/µL (*p* = 0.03). Values above 100% may be a reflection of increased metabolic activity relative to control as measured by the MTT assay. * Statistical significance defined as *p* < 0.05. The red dashed line indicates 100% of cell viability.

To assess whether exposure duration to different *C. cyminum* extracts affected HMEC-1 cell viability, we compared the results at 24 h and 48 h. The findings, depicted in Figure 2, indicate variations in endothelial cell viability over these time periods for specific extracts and concentrations.

For the aqueous extract, a significant difference was observed at 1.6 µg/µL (*p* = 0.013) (Figure 2a). In the methanolic extract, differences in cell viability were noted at 1.6 µg/µL and 6.4 µg/µL, with *p*-values of 0.007 and 0.004, respectively (Figure 2b). The acetonic extract did not show statistically significant differences in cell viability between 24 h and 48 h (Figure 2c). For the hexane extract, a significant difference was found at 6.4 µg/µL (*p* = 0.03) (Figure 2d).

In addition to the statistical differences observed between exposure times, the magnitude of the changes in HMEC-1 cell viability varied notably depending on the extract and concentration (Figure 2). For the aqueous extract, the time-dependent difference detected at 1.6 µg/µL corresponded to a modest reduction in cell viability, with values decreasing from approximately 95% at 24 h to about 88–90% at 48 h, representing a reduction of roughly 5–7%. In contrast, the methanolic extract exhibited the most pronounced time-dependent effects. At 1.6 µg/µL, cell viability decreased from approximately 65% at 24 h to nearly 45% at 48 h, corresponding to an additional reduction of around 20%. At 6.4 µg/µL, viability declined from about 45% at 24 h to approximately 40% at 48 h, indicating an overall reduction of nearly 55–60% relative to the untreated control.

For the acetonic extract, although no statistically significant differences between 24 and 48 h were observed, high concentrations were associated with marked cytotoxicity. At 6.4 µg/µL, cell viability was reduced to approximately 40% at both time points, representing a decrease of nearly 60% compared with control conditions. Finally, for the hexane extract, the significant time-dependent effect observed at 6.4 µg/µL was associated with a decrease in cell viability from approximately 80% at 24 h to about 75% at 48 h, corresponding to a reduction of approximately 20–25% relative to the control.

At lower concentrations, several *C. cyminum* extracts induced cell viability values exceeding 100% at both 24 and 48 h, particularly in the methanolic and acetonic groups, suggesting preserved or slightly increased metabolic activity relative to control conditions. It should be noted that values above 100% obtained in the MTT assay do not necessarily indicate an increase in cell number, as this assay primarily reflects mitochondrial metabolic activity rather than cell proliferation [47]. Accordingly, these responses should be interpreted as indicative of transient metabolic stimulation under specific conditions or intrinsic assay variability, rather than enhanced endothelial cell proliferation [48].

### 3.3. IC_50_ Analysis of Endothelial Cytotoxicity Induced by C. cyminum Extracts

**Figure 3:**
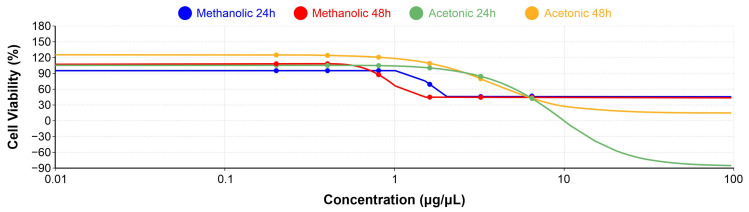
IC_50_ results for methanolic and acetonic extracts. The graph displays the IC_50_ values at 24 and 48 h post-treatment, indicating the concentrations required to achieve a 50% reduction in cell viability for each extract. The methanolic extract shows IC_50_ values of 1.55 µg/µL at 24 h and 0.91 µg/µL at 48 h. The acetonic extract exhibits IC_50_ values of 8.94 µg/µL at 24 h and 3.82 µg/µL at 48 h. Note that IC_50_ values for the aqueous and hexane extracts are not included due to lack of measurable cell inhibition.

The half maximal inhibitory concentration (IC_50_) was determined using the online AAT Bioquest Inc. Quest Graph™ LD50 Calculator [43], adapted to calculate inhibitory concentrations from cell viability data. The IC_50_ represents the concentration of a substance required to reduce cell viability by 50% relative to the untreated control. At 24 h of exposure, the IC_50_ values were 1.55 µg/µL for the methanolic extract and 8.94 µg/µL for the acetonic extract (Figure 3).

After 48 h, these values decreased to 0.91 µg/µL and 3.82 µg/µL for the methanolic and acetonic extracts, respectively. IC_50_ values could not be determined for the aqueous and hexane extracts using this approach due to the absence of sufficient inhibitory effects within the tested concentration range.

### 3.4. Evaluation of Effect of C. cyminum Extracts on Endothelial Cell Migration

To evaluate whether *C. cyminum* extracts influenced HMEC-1 cell migration, a classical wound-healing assay was performed. Briefly, a confluent HMEC-1 cell monolayer was mechanically scratched and subsequently exposed to 0.8 µg/µL and 1.6 µg/µL of each extract. Wound closure was monitored over time, and the extent of wound closure was quantified at defined time points.

**Figure 4:**
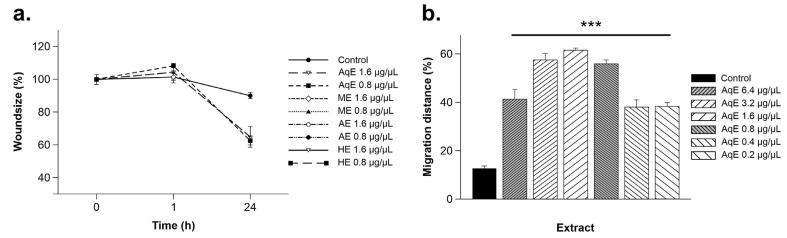
Effect of the *C. cyminum* extracts on the endothelial cell migration. Human micro-endothelial cells (HMEC-1) were cultured and exposed to different concentrations of *C. cyminum* extracts. After incubation at 37 °C, during different intervals of times, the wound size was measured and plotted (**a**). For methanolic, acetonic, and hexane extracts the cell migration could be measured only at 1 h because the toxicity of each extract and at 24 h, the percentage of the wound size was below in the aqueous extract to that observed in the control. (**b**) At the 24 h, for the aqueous extract, the distance of migration of HMEC-1 exposed to six different concentrations (0.2 µg/µL–6.4 µg/µL) was calculated and compared against the control. The comparisons were carried out by two-way ANOVA using the control as reference. *** Indicate *p* < 0.001. Figure displays the results of the quantification of three independent experiments.

As shown in Figure 4a, the effects of *C. cyminum* extracts on HMEC-1 cell migration were assessed at 0, 1, and 24 h after treatment. These time points were selected based on viability and IC_50_ data to evaluate early and sustained effects on endothelial migration under sublethal exposure conditions. Compared with the untreated control, a significant reduction in wound closure was observed at 24 h for all extracts and concentrations evaluated (*p* < 0.05), indicating impaired migratory capacity. Given that no cytotoxic effects were observed for the aqueous extract at 0.8 µg/µL and 1.6 µg/µL after 24 h of exposure, the migratory response of HMEC-1 cells was further evaluated by expanding the concentration range of the aqueous extract (0.2–6.4 µg/µL) under non-lethal conditions. The comparative analysis of HMEC-1 migration distance relative to the control is presented in Figure 4b. In contrast to the other extracts, treatment with the aqueous extract significantly enhanced HMEC-1 cell migration at all concentrations tested (*p* < 0.001). Notably, the observed increase in cell migration was not concentration-dependent.

### 3.5. Effects of Cuminum cyminum Extract Treatments on Gene Expression in HMEC-1 Cells

**Figure 5:**
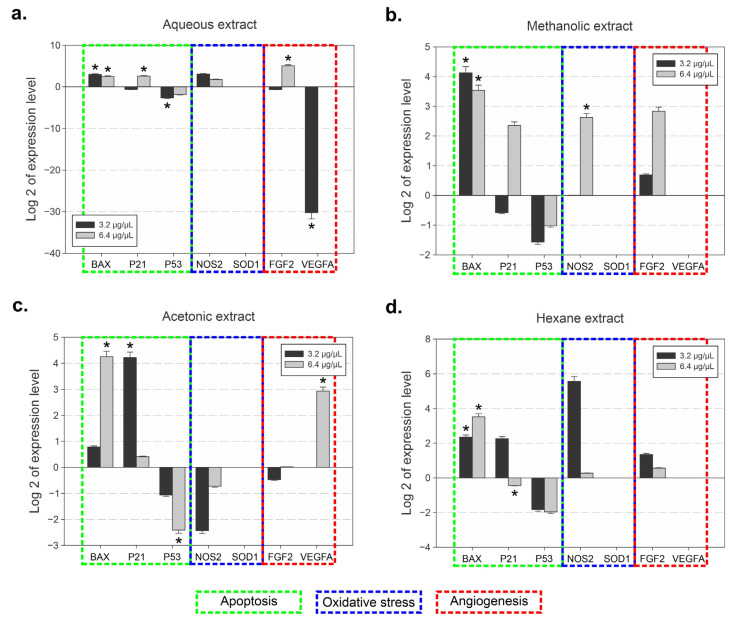
Solvent-dependent modulation of apoptosis-, oxidative stress-, and angiogenesis-related gene expression in HMEC-1 cells treated with *C. cyminum* extracts. Relative gene expression of apoptosis-related (*BAX, P21, P53*), oxidative stress-related (*NOS2, SOD1*), and angiogenesis-related (*FGF2, VEGFA*) genes in HMEC-1 cells treated with aqueous (**a**), methanolic (**b**), acetonic (**c**), and hexane (**d**) extracts of *C. cyminum* at concentrations of 3.2 and 6.4 µg/µL. Gene expression levels were normalized to the endogenous control (*GAPDH*) and expressed as log_2_ fold change relative to untreated cells used as calibrators. Data are presented as mean ± SD. Asterisks indicate statistically significant differences compared with untreated control cells (*p* < 0.05). Green dashed boxes denote apoptosis-related genes, blue dashed boxes indicate oxidative stress-related genes, and red dashed boxes correspond to angiogenesis-related genes.

To assess the effect of *C. cyminum* extracts on endothelial gene expression, HMEC-1 cells were exposed to aqueous, methanolic, acetonic, and hexane extracts at concentrations of 0.8, 1.6, 3.2, and 6.4 µg/µL for 48 h. Transcriptional changes were evaluated by qRT-PCR for genes associated with apoptosis (*BAX, P53, P21*), oxidative stress response (*SOD1, NOS2*), and angiogenesis (*FGF2, VEGFA*), using *GAPDH* as the endogenous reference. Extract- and concentration-dependent modulation of gene expression was observed, and the results are summarized in Figure 5.

Treatment with the aqueous extract resulted in significant modulation of apoptosis-related genes (Figure 5a). *BAX* expression showed positive log_2_ fold changes at both 3.2 µg/µL (2.98 ± 0.15, *p* < 0.05) and 6.4 µg/µL (2.50 ± 0.13, *p* < 0.05). *P21* exhibited a modest but significant increase at 6.4 µg/µL (2.56 ± 0.13, *p* < 0.05), whereas *P53* showed significant negative log_2_ fold changes at 3.2 µg/µL (−2.64 ± 0.13, *p* < 0.05). In the OS pathway, *NOS2* expression was significantly increased at both 3.2 µg/µL (3.06 ± 0.15, *p* < 0.05) and 6.4 µg/µL (1.76 ± 0.09, *p* < 0.05), while *SOD1* expression was not detected. Regarding angiogenesis-related genes, *FGF2* was significantly upregulated at 6.4 µg/µL (5.05 ± 0.25, *p* < 0.05), whereas *VEGFA* exhibited a pronounced negative log_2_ fold change at 3.2 µg/µL (−30.24 ± 1.51, *p* < 0.05).

In cells treated with the methanolic extract (Figure 5b), *BAX* expression was significantly increased at both concentrations (4.13 ± 0.21 at 3.2 µg/µL and 3.54 ± 0.18 at 6.4 µg/µL; *p* < 0.05). *P21* showed a significant decrease at 3.2 µg/µL (−0.58 ± 0.03) and a significant increase at 6.4 µg/µL (2.36 ± 0.12), while *P53* was significantly downregulated at both concentrations. *NOS2* expression remained close to baseline at 3.2 µg/µL but was significantly upregulated at 6.4 µg/µL (2.62 ± 0.13). In the angiogenesis-related group, *FGF2* showed significant positive log_2_ fold changes at both concentrations (*p* < 0.05), whereas *VEGFA* expression was not detected.

As shown in Figure 5c, the acetonic extract induced differential expression patterns. *BAX* expression increased significantly at 6.4 µg/µL (4.25 ± 0.21). *P21* was significantly upregulated at 3.2 µg/µL (4.22 ± 0.21) but significantly reduced at 6.4 µg/µL (0.41 ± 0.02). *P53* expression showed significant negative log_2_ fold changes at both concentrations, with a greater reduction at 6.4 µg/µL (−2.41 ± 0.12). In the OS pathway, *NOS2* expression was significantly downregulated at both concentrations (*p* < 0.05). In contrast, *VEGFA* expression was significantly increased at 6.4 µg/µL (2.94 ± 0.15, *p* < 0.05), while *FGF2* remained close to baseline.

Finally, treatment with the hexane extract (Figure 5d) resulted in significant positive log_2_ fold changes in *BAX* at both 3.2 µg/µL (2.36 ± 0.12) and 6.4 µg/µL (3.52 ± 0.18). *P21* expression was significantly increased at 3.2 µg/µL (2.26 ± 0.11) but significantly decreased at 6.4 µg/µL (−0.45 ± 0.02). *P53* exhibited significant negative log_2_ fold changes at both concentrations. A marked induction of *NOS2* expression was observed at 3.2 µg/µL (5.57 ± 0.28), whereas expression levels approached baseline at 6.4 µg/µL. *FGF2* showed significant positive log_2_ fold changes at both concentrations (*p* < 0.05), while *VEGFA* expression was not detected.

### 3.6. Evaluation of Effect of Cuminum cyminum Extracts on the Morphology of HMEC-1

**Figure 6:**
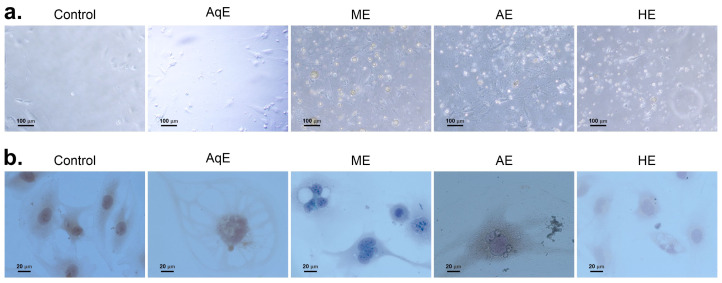
Cytologic evaluation of the *Cuminum cyminum* extracts on the endothelial cells. Human micro-endothelial cells (HMEC-1) were cultured and exposed to 0.8 µg/µL of *Cuminum cyminum* aqueous and methanolic extracts. After incubation at 37 °C, during 48 h, cells were observed under light microscopic observations (**a**) or stained with Papanicolaou staining for evaluating morphologic changes (**b**). The presence of methanolic, acetonic, and hexane extracts induced cell death. Abbreviations: AqE: aqueous extract; ME: methanolic extract; AE: acetonic extract; HE: hexane extract.

To evaluate the morphological changes induced by exposure of HMEC-1 cells *to C. cyminum* extracts, the HMEC-1 cells were cultured to 50% confluence on chamber slides and exposed to 0.8 µg/µL for 24 h. After exposure, the cultures were observed directly under light microscopy. To contrast the cell membrane, cytoplasm, and nucleus morphology, the slides were stained with Papanicolaou stain. The results are shown in Figure 6.

The main cytological observations were as follows. In control HMEC-1 cells, the background was clean (Figure 6a). Most cells appeared mature, with a central oval nucleus and a small amount of cytoplasm with well-defined borders, often showing close cell–cell contact. Chromatin was finely granular and evenly distributed (Figure 6b).

Compared with control cells, HMEC-1 cells exposed to the aqueous extract of *C. cyminum* exhibited cytological alterations. Some cells showed dense, eosinophilic cytoplasm with degenerative changes, including the presence of microvacuoles. Nuclear contraction and chromatin condensation (pyknosis) were also observed, consistent with necrotic changes (Figure 6b).

Cells exposed to the methanolic extract presented a relatively clean background with numerous cells and few cellular projections (Figure 6a). Most cells appeared mature but displayed irregular cell membrane borders and occasional cytoplasmic vacuolization. Nuclei were round to oval with thick, coarsely granular chromatin (Figure 6b). In addition, numerous multinucleated cells were observed, and some exhibited nuclear alterations such as pyknosis, hyperchromasia, and nuclear shrinkage. The cytoplasm of these cells was dense and eosinophilic, similar to that observed in cells treated with the aqueous extract. Exposure of HMEC-1 cells to the acetonic extract resulted in a background with abundant cellular debris (Figure 6a, b). Numerous cells exhibited clear cytoplasm and one or two nuclei. Nuclear membrane irregularities, including indentations, were frequently observed, and some cells displayed karyomegaly (Figure 6b). Treatment with the hexane extract also produced a background characterized by marked cellular debris. Some endothelial cells exhibited irregular nuclear membrane contours, binucleation, and karyomegaly. In a smaller proportion of cells, typical mitotic figures, including anaphase, were observed. Additionally, mild cytoplasmic vacuolization was detected in a limited number of cells (Figure 6b).

The in vitro findings revealed a consistent pattern characterized by preserved endothelial viability in the presence of functional, molecular, and cytomorphological alterations, particularly in cells exposed to aqueous extracts at non-cytotoxic concentrations. This pattern, hereafter referred to as sublethal endothelial injury (SEI), was observed across multiple experimental endpoints.

### 3.7. Biological Activities of the Major Constituents of Cuminum cyminum

With the aim of identifying biologically relevant phytochemicals reported across different solvent extracts of *C. cyminum*, a literature-based search was conducted to compile and systematize compounds identified using diverse analytical platforms, including GC–MS, LC–MS, and HPLC. This approach allowed the integration of both quantitative and qualitative evidence across aqueous, methanolic, acetonic, hexane extracts, and essential oil preparations, highlighting solvent-dependent trends in phytochemical composition (Supplementary Table S1). The analysis revealed a clear polarity-driven segregation of chemical classes. Non-polar extracts, particularly essential oil and hexane extracts were consistently enriched in volatile and lipophilic constituents, with aromatic aldehydes and monoterpenes representing the dominant phytochemical groups. Cuminaldehyde emerged as the most consistently reported major compound across non-polar and semi-polar extracts, frequently accompanied by *γ*-terpinene and *p*-cymene as principal monoterpenes, confirming their central role in cumin aroma and bioactivity [5,8,49]. Additional terpenes such as *α*-pinene, *β*-pinene, limonene, and caryophyllene oxide were repeatedly identified in essential oil and hexane extracts, although their reported abundances varied considerably depending on geographic origin, extraction method, and analytical conditions.

Methanolic and ethanolic extracts displayed a mixed phytochemical profile, retaining several volatile constituents, most notably cuminaldehyde and *p*-cymene, while also incorporating less volatile lipophilic compounds, including fatty acids and their esters. Oleic acid and methyl oleate were recurrently reported in methanolic extracts, reflecting the ability of intermediate-polarity solvents to extract both aromatic and lipid-derived metabolites [50]. Acetonic extracts showed an intermediate and less comprehensively characterized profile, sharing aromatic aldehydes and monoterpenes with methanolic and hexane extracts, but with fewer studies reporting detailed compositional data. In contrast, polar extracts were dominated by phenolic and polyphenolic constituents. Aqueous extracts consistently exhibited a high diversity of hydrophilic metabolites, particularly phenolic acids and flavonoids, although individual compound quantification was limited. Ellagic acid and rutin were the only phenolics quantified at the individual level in aqueous extracts by HPLC, while other phenolic acids, including gallic, caffeic, *p*-coumaric, and ferulic acids, were repeatedly identified across primary studies and reviews without reported concentrations [51,52,53]. Flavonoids such as quercetin, apigenin, and luteolin were predominantly reported in methanolic and ethanolic extracts, supporting their classification as key semi-polar constituents. Tannins and saponins were consistently reported through qualitative phytochemical screening in aqueous and methanolic extracts, aligning with the documented antimicrobial, antioxidant, and metabolic effects of polar cumin preparations [6,53].

**Figure 7:**
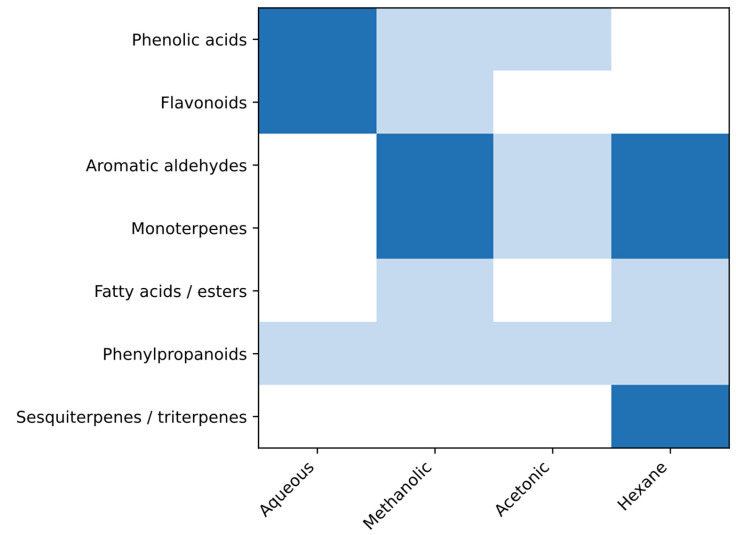
Differential phytochemical group enrichment across aqueous, methanolic, acetonic, and hexane extracts of *Cuminum cyminum*. The heatmap summarizes the relative enrichment of major phytochemical groups in *C. cyminum* extracts based on literature-reported compositional data from primary research articles and review studies. The analysis includes phenolic acids, flavonoids, aromatic aldehydes, monoterpenes, fatty acids/esters, phenylpropanoids, and sesquiterpenes/triterpenes identified across different extraction solvents. Enrichment levels were assigned using a semi-quantitative, evidence-based scale reflecting both consistency of reporting and availability of quantitative data: 0 (white) indicates not reported or insufficient evidence, 1 (light blue) indicates repeated identification without individual quantitative values, and 2 (dark blue) indicates compounds reported as major constituents or with quantitative abundance data.

To visualize and compare the dominant phytochemical patterns associated with each *C. cyminum* extract, a phytochemical group enrichment analysis was performed using a semi-quantitative, literature-based scoring approach derived from compound recurrence and, when available, reported abundance (Figure 7). Aqueous extracts were predominantly enriched in polar phytochemical groups, particularly phenolic acids and flavonoids, including gallic, caffeic, *p*-coumaric, ferulic, and ellagic acids, as well as flavonoids such as rutin, quercetin, apigenin, and luteolin, together with tannins and saponins consistently identified through qualitative screening [6,51,52,53].

Methanolic extracts displayed a mixed enrichment profile, characterized by aromatic aldehydes and monoterpenes, most notably cuminaldehyde, *p*-cymene, *γ*-terpinene, and limonene, alongside fatty acids and their esters, reflecting the broad extraction capacity of intermediate-polarity solvents [25,49,50]. Acetonic extracts showed aromatic aldehydes and terpenoids with methanolic and hexane extracts but with fewer consistently reported phenolic constituents [2,53]. In contrast, hexane extracts were strongly enriched in non-polar phytochemical groups, including monoterpenes, aromatic aldehydes, phenylpropanoids, and highly lipophilic constituents such as sesquiterpenes and triterpenes, with cuminaldehyde, D-carvone, apiol, squalene, and caryophyllene oxide repeatedly reported as representative markers [5,8,49].

### 3.8. Selection of Cumin-Derived Compounds for In Silico Biological Activity Prediction

To prioritize compounds for in silico biological activity prediction, a tiered selection strategy was applied integrating phytochemical prevalence derived from the literature (Supplementary Table S1), extract-specific phytochemical group enrichment patterns (Figure 7), and documented biological relevance to endothelial-related endpoints evaluated in this study. The final core set included nine compounds representative of the dominant chemical classes identified across aqueous, methanolic, acetonic, and hexane extracts of *C. cyminum*. This selection encompassed major volatile and semi-volatile constituents (cuminaldehyde, *p*-cymene, and *γ*-terpinene), which are consistently reported as abundant components of non-polar and semi-polar cumin extracts. In parallel, key phenolic acids and flavonoids (gallic, caffeic, and ferulic acids, and quercetin) were selected based on their recurrent identification in polar extracts and their reported anti-inflammatory and vasoprotective activities. Representative phytosterols (*β*-sitosterol and stigmasterol) were also included due to their association with lipophilic extract fractions and their well-documented membrane-stabilizing and endothelial-modulatory bioactivities. Collectively, this compound set provided broad chemical class representation while minimizing redundancy and overrepresentation, thereby supporting a focused and biologically relevant in silico prediction analysis.

### 3.9. In Silico Prediction of Endothelial-Related Biological Activities

**Figure 8:**
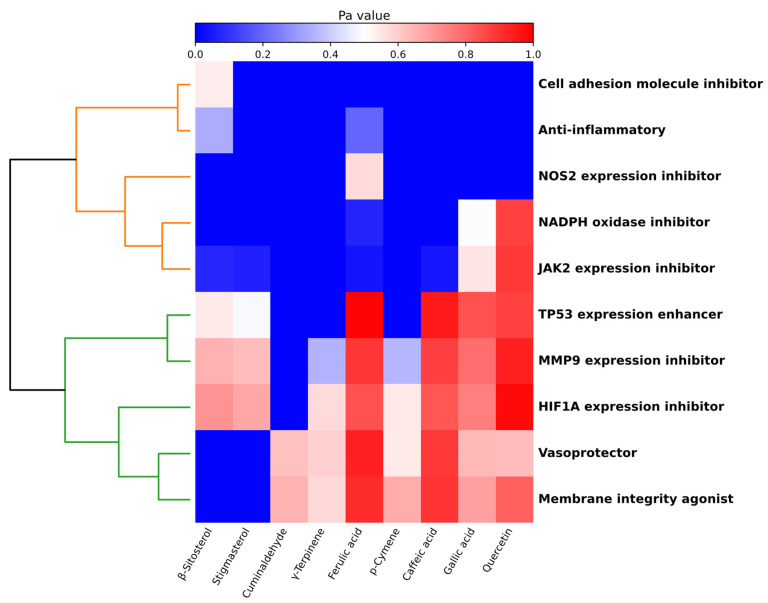
Hierarchical heatmap showing the activity probability (Pa) of bioactive compounds from *Cuminum cyminum* and different biological activities. The blue–white–red color gradient represents increasing Pa values, where deep blue indicates absence of activity or Pa < 0.5, and red indicates a higher probability of association. Biological activities were grouped using hierarchical clustering based on the similarity of their functional profiles and are displayed in inverted order to highlight those with higher overall signal at the bottom. The dendrogram illustrates functional relationships among biological activities, where branch colors denote distinct clusters of related biological functions. Compounds are displayed in a predefined order to facilitate comparative interpretation of activity patterns.

To explore potential molecular contributors to the observed in vitro endothelial responses, an in silico analysis of selected phytochemical constituents was performed using the PASS Online software (v2.0). All PASS-based predictions were employed as supportive, hypothesis-generating. The analysis revealed distinct, compound-specific probability profiles across multiple biological activities relevant to endothelial structure, redox balance, and vascular function (Figure 8). Overall, phenolic acids and flavonoids exhibited the highest and most consistent Pa values across several endothelial-related functional axes, whereas volatile terpenoids and phytosterols displayed more selective and pathway-specific prediction patterns. Enhancement of *TP53* expression was one of the most consistently predicted activities across the compound set, with high Pa values observed for phenolic acids such as gallic, caffeic, and ferulic acids, as well as for the flavonoid quercetin. A similar trend was observed for inhibition of *HIF1A* expression, where phenolic compounds again showed strong prediction probabilities, supporting a potential role in the modulation of hypoxia-related endothelial signaling. In contrast, volatile terpenoids such as *p*-cymene and *γ*-terpinene displayed moderate and more restricted Pa values for these transcriptional endpoints.

Activities associated with endothelial migration and extracellular matrix remodeling, represented by MMP9 expression inhibition, were predicted for multiple compounds, with phenolic acids, particularly caffeic and ferulic acids, showing the highest probabilities. Redox-related activities exhibited a narrower distribution: NADPH oxidase inhibition was primarily predicted for quercetin, whereas *NOS2* expression inhibition was associated with selected phenolic acids, suggesting a compound-dependent modulation of oxidative and nitrosative stress pathways. Membrane-related activities constituted one of the most prominent predicted functional categories. Membrane integrity agonist activity showed high Pa values for gallic, caffeic, and ferulic acids, as well as for quercetin, consistent with their reported interactions with lipid bilayers and endothelial barrier function. Vasoprotective activity was also predicted for several phenolic compounds and selected terpenoids, indicating potential contributions to vascular homeostasis beyond antioxidant effects. Phytosterols displayed a distinct prediction profile, with β-sitosterol and stigmasterol showing moderate Pa values for selected endothelial-related activities, particularly those associated with membrane stabilization and signaling modulation, rather than transcriptional regulation.

## 4. Discussion

*C. cyminum* has been extensively investigated for its antimicrobial, antioxidant, and anticancer properties. However, most of the available evidence is derived from crude toxicity assays, antioxidant capacity tests, or cancer cell models, with limited attention to endothelial biology, dose-dependent cytotoxic thresholds, and functional outcomes beyond cell viability. In this context, the present study extends current knowledge by providing an integrative characterization of extract-dependent endothelial responses, combining cytotoxicity, migration, gene expression, cytomorphology, and in silico predictions in the experimental design.

Our results demonstrate that methanolic and acetonic extracts induce marked endothelial cytotoxicity, accompanied by time-dependent reductions in IC_50_ values, whereas aqueous extracts exhibit minimal cytotoxic effects at equivalent concentrations. This distinction is particularly relevant given the high sensitivity of endothelial cells to redox imbalance and membrane perturbation. Notably, the IC_50_ value observed for methanolic extracts at 48 h (0.91 µg/µL) falls within concentration ranges commonly employed in phytochemical bioassays, suggesting that endothelial toxicity may be underestimated in studies that do not explicitly include vascular cell types. This represents a relevant contribution, as endothelial dysfunction is a central event in chronic inflammatory disorders and has been linked to disease progression and complications in metabolic and vascular diseases [15].

The relevance of these findings becomes clearer when considered alongside the traditional medicinal uses of *C. cyminum*, which include treatment of ulcers and boils, reduction in inflammation, diuretic effects, and antihypertensive activity [11,12,13]. Previous in vivo studies have shown that *C. cyminum* seeds improve endothelial function and attenuate inflammation and OS in hypertensive rat models [54]. In our study, HMEC-1 cells exposed to the aqueous extract did not show cytotoxic effects at 24 h and maintained viability comparable to control cells at most tested concentrations. Although a modest reduction in viability was observed between 24 and 48 h at lower concentrations (0.8–1.6 µg/µL), viability at higher concentrations remained similar to control levels (*p* > 0.05). In contrast, methanolic extracts produced a gradual reduction in viability over the range of 1.6–6.4 µg/µL at both time points, while acetonic extracts induced toxicity primarily at the highest concentration tested (6.4 µg/µL). These observations align with previous reports showing concentration-dependent effects of cumin-derived extracts across different cell types. For example, L929 fibroblasts exposed to *C. cyminum* essential oil retained viability above 70% at concentrations between 2 and 8 mg/mL, whereas cytotoxicity was observed at 20 mg/mL [55]. Similarly, tenocytes and L929 fibroblasts treated with *C. cyminum* nanoparticles maintained stable viability across a concentration range of 20–80 µg, with maximal viability at higher concentrations [42]. However, these studies did not address endothelial-specific responses, highlighting the relevance of the present endothelial-focused evaluation.

Beyond viability, a key contribution of this work is the demonstration that *C. cyminum* extracts exert extract-specific effects on endothelial migration, a functional endpoint that has not been previously evaluated in this context. At sub-cytotoxic concentrations (0.8 and 1.6 µg/µL), HMEC-1 migration exceeded that of control cells, with the effect being most pronounced for the aqueous extract across a broader concentration range (0.4–6.4 µg/µL). Although no prior studies have directly assessed the impact of cumin extracts on endothelial migration, these findings are consistent with mechanistic insights from in vivo models. Kalaivani et al. reported that chronic administration of aqueous *C. cyminum* extract improved plasma NO levels, reduced systolic blood pressure, and upregulated endothelial nitric oxide synthase (eNOS) expression in carotid arteries of rats with hypertension, while reversing pathological vascular remodeling [54]. The enhanced endothelial migration observed in our in vitro model may therefore reflect a functionally adaptive or pro-reparative endothelial phenotype.

To place these functional outcomes in a mechanistic context, phytochemical group enrichment and in silico prediction analyses were integrated into the interpretation of the experimental findings. Although comprehensive chemical characterization of the extracts was not performed in this study, the major phytochemical classes associated with each extraction strategy, particularly phenolic acids (gallic, caffeic, and ferulic acids), flavonoids (quercetin), volatile terpenoids (cuminaldehyde, *p*-cymene, and *γ*-terpinene), and phytosterols (β-sitosterol and stigmasterol)—have been repeatedly identified and structurally characterized in *Cuminum cyminum* extracts by independent studies using GC–MS, LC–MS, and HPLC approaches [2,4,5,8,10,53]. Importantly, these compounds have also been experimentally associated with biological activities relevant to endothelial function, including modulation of OS, inflammatory signaling, cell cycle regulation, and membrane dynamics, across diverse experimental models [7,25,56]. In this context, PASS Online predictions were used as a hypothesis-generating tool to prioritize biologically plausible activities rather than to assign definitive effects. Phenolic acids and flavonoids, predominantly associated with polar and semi-polar extracts, were predicted to modulate membrane integrity, TP53-related signaling, JAK2-associated pathways, and vasoprotective responses, which is consistent with reported antioxidant, anti-inflammatory, and endothelial-protective properties of gallic, caffeic, and ferulic acids, as well as quercetin [7,56]. These predictions are coherent with the extract- and dose-dependent preservation of endothelial viability and migratory capacity, as well as with the selective transcriptional modulation observed experimentally, including upregulation of *FGF2* at lower concentrations and moderate induction of *NOS2* in methanolic and hexane extracts, rather than a uniform pro-survival response across all conditions. However, these in silico predictions should be interpreted cautiously and considered as complementary to the experimental findings rather than direct mechanistic proof.

Conversely, terpenoid-rich and lipophilic fractions, characteristic of methanolic, acetonic, and hexane extracts, were associated with predicted activities linked to OS signaling, hypoxia-related pathways (*HIF1A*), and extracellular matrix remodeling (MMP9), consistent with previous reports indicating that cumin volatile and lipophilic fractions can exert concentration-dependent cytotoxic and pro-oxidant effects [2,5,53]. This profile provides a hypothesis-generating background to interpret the concurrent induction of pro-apoptotic markers (*BAX* upregulation), suppression of cell cycle regulators (*P53* downregulation), and loss of endothelial integrity observed at higher extract concentrations, particularly in acetonic and methanolic fractions, with hexane extracts exhibiting reduced viability only at the highest concentration tested. Notably, the divergence between angiogenic signaling (*FGF2, VEGFA*) and oxidative/apoptotic responses highlights a narrow functional window in which endothelial activation is preserved before cytotoxic signaling predominates, supporting the notion that cumin-derived phytochemical mixtures exert context- and dose-dependent effects on endothelial homeostasis [5,53].

The molecular and cytomorphological analyses further refined the interpretation of these functional outcomes by revealing extract-specific endothelial states that extend beyond viability measurements. In cells treated with aqueous extracts, subtle cytoplasmic microvacuolization and mild membrane irregularities were accompanied by upregulation of *FGF2* and moderate induction of *NOS2* at non-cytotoxic concentrations, without pronounced activation of pro-apoptotic signaling, indicating a viable but functionally altered endothelial state compatible with early adaptive stress–response or remodeling processes rather than overt injury (Table 3). This phenotype is consistent with the reported endothelial-modulatory and membrane-stabilizing properties of phenolic acids and flavonoids commonly identified in aqueous cumin extracts [5,10,53].

In contrast, exposure to methanolic and acetonic extracts resulted in pronounced nuclear abnormalities, including pyknosis and hyperchromasia, which coincided with strong upregulation of the pro-apoptotic gene *BAX* and downregulation of *TP53*, supporting apoptosis-associated mechanisms. These alterations were accompanied by reduced viability and impaired migratory capacity at higher concentrations, consistent with concentration-dependent cytotoxic effects previously reported for terpenoid-rich and semi-polar cumin fractions [2,5,53]. Hexane extracts displayed a distinct profile characterized by marked *NOS2* upregulation, dysregulation of cell-cycle-associated genes, and severe morphological disruption, including cellular debris and atypical mitotic figures, supporting a state of overt endothelial injury associated with oxidative and nitrosative stress [5,56].

The most evident morphological alterations were observed in cells exposed to non-polar extracts, particularly hexane at higher concentrations, where extensive cellular debris, nuclear membrane irregularities, karyomegaly, and atypical mitotic figures were evident. These features were consistent with marked induction of *NOS2* expression and dysregulation of cell cycle-related genes, supporting a shift toward oxidative and nitrosative stress-mediated endothelial injury rather than purely apoptotic mechanisms [5,56]. Importantly, these alterations occurred in parallel with a reduction in cell viability only at the highest concentration tested, reinforcing the notion that structural and molecular damage precede overt cytotoxic collapse. Additionally, these alterations may be influenced by the combined effects of lipophilic phytochemicals and non-organic constituents, as trace elements such as bromine, arsenic, and antimony have been previously detected in *C. cyminum* seeds and extracts, particularly in non-polar fractions [5,57]. Although the contribution of these elements was not directly assessed in the present study, their potential involvement in amplifying oxidative stress and genotoxic signaling cannot be excluded.

**Figure 9:**
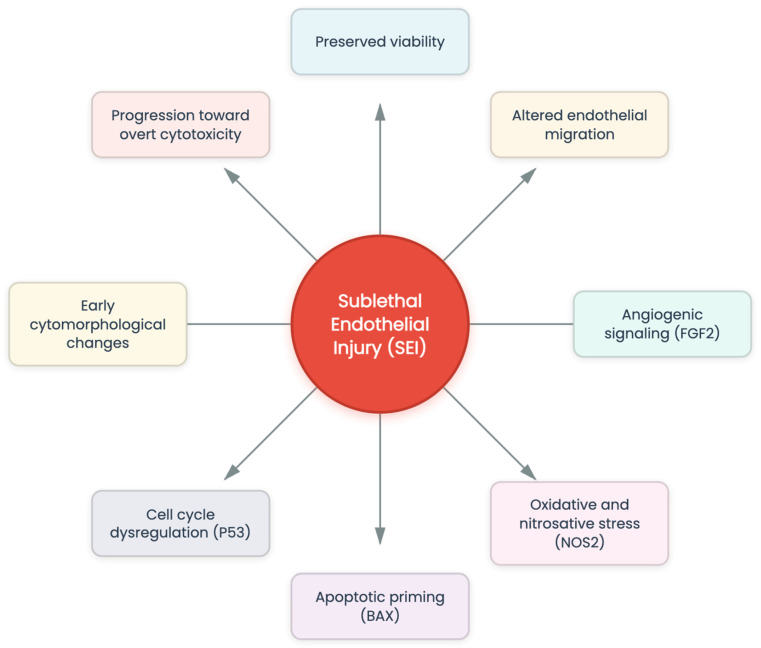
Hallmarks of sublethal endothelial injury. Schematic representation of the intermediate endothelial state defined in this study as sublethal endothelial injury (SEI). SEI is characterized by preserved or moderately reduced cell viability accompanied by early cytomorphological alterations, altered endothelial migration, and coordinated molecular changes, including activation of angiogenic signaling (*FGF2*), oxidative and nitrosative stress (*NOS2*), apoptotic priming (*BAX*), and dysregulation of cell cycle control (*TP53*). This state represents a transitional condition between physiological endothelial activation and progression toward overt cytotoxicity and integrates the functional, transcriptional, and morphological findings obtained in the present study.

Our results support the identification of a biological state characterized by preserved or moderately reduced endothelial viability accompanied by functional, molecular, and morphological alterations, which we propose as sublethal endothelial injury (SEI). SEI represents an intermediate condition between physiological endothelial activation and overt cytotoxic injury and provides a useful conceptual framework to distinguish non-lethal yet biologically relevant endothelial perturbations from irreversible damage [7,53]. Recognizing SEI is particularly relevant when evaluating complex plant-derived extracts, where reliance on viability assays alone may obscure early vascular dysfunction and stress-adaptive remodeling processes (Figure 9).

The conceptual model depicted in Figure 9 integrates the functional, molecular, and morphological findings of this study into a unified context of SEI. Rather than relying solely on cytotoxicity as an endpoint, this model emphasizes that endothelial perturbation may manifest through coordinated alterations in migration dynamics, redox-sensitive signaling, angiogenic responses, apoptotic priming, and cell-cycle regulation. Consistent with current vascular biology and toxicology concepts, endothelial dysfunction is increasingly recognized to arise from early functional and molecular disturbances that can precede irreversible injury and may occur without marked cell death [17,58]. In support of this perspective, function-oriented endothelial readouts, including migration and wound-healing behavior as well as barrier-related alterations, are widely used to capture early vasculotoxicity beyond viability-based screening [59,60,61]. This distinction is particularly relevant because commonly used metabolic viability assays may remain preserved despite biologically meaningful cellular dysregulation, underscoring that viability alone is an incomplete proxy for endothelial integrity [47]. Within this context, the SEI model provides an integrative interpretation of these early endothelial alterations, extending beyond isolated pathway analyses to describe a coordinated and biologically meaningful endothelial state.

In summary, our results showed that *C. cyminum* extracts exert divergent, extract-dependent effects on endothelial cells, ranging from cytotoxic and anti-migratory responses to non-lethal, pro-migratory phenotypes. By integrating functional assays, molecular profiling, cytomorphology, and in silico predictions, this study contributes new insight into the vascular relevance and safety considerations of cumin-derived phytochemicals, an aspect that has been largely overlooked in previous research. Importantly, the effects described here were observed using concentrated extracts under controlled in vitro conditions and should not be directly extrapolated to typical dietary exposure associated with culinary use of cumin. Rather, these findings highlight the need to consider endothelial-specific functional endpoints when evaluating the safety of concentrated or supplemental plant-derived extracts. Additionally, the lack of in vivo validation, the use of crude extracts without compound-level separation, and the potential influence of solvent-dependent extraction represent important limitations that warrant further investigation in future studies.

### Limitations and Future Directions

Some study limitations must be acknowledged: (1) the absence of quantitative, compound-level chemical characterization of the tested extracts (e.g., HPLC or GC–MS profiling) and the use of crude extracts preclude precise attribution of the observed effects to individual constituents. Additionally, solvent-dependent extraction may influence both the qualitative and quantitative composition of the extracts, potentially contributing to differential endothelial responses. Future work incorporating HPLC/GC–MS-based quantification, extract fractionation, and/or purified analytes will be essential to validate structure–activity relationships and to directly correlate specific phytochemicals with endothelial functional outcomes. (2) Gene expression analyses were used as an exploratory mechanistic approach; however, validation at the protein level (e.g., Western blotting or immunofluorescence) will be required in future studies to confirm the functional relevance of these molecular alterations. (3) Additionally, the findings are limited to an in vitro endothelial model and should be interpreted cautiously in the context of in vivo vascular physiology. Future studies should focus on redox-sensitive signaling pathways (e.g., NF-κB, Nrf2), endothelial barrier function, and in vivo angiogenesis models to determine whether the extract-specific effects observed here translate into physiological or pathological outcomes.

## 5. Conclusions

The biological impact of *C. cyminum* extracts is strongly dependent on extraction solvent and exposure duration. This study demonstrated that *C. cyminum* extracts elicit divergent endothelial responses that span from SEI to overt cytotoxic damage. Aqueous extracts exhibited a favorable endothelial profile at non-cytotoxic concentrations, maintaining cell viability comparable to untreated controls (*p* > 0.05) across most tested doses while significantly enhancing endothelial migration over a broad concentration range (0.4–6.4 µg/µL, *p* < 0.001). This functional response was accompanied by modulation of angiogenesis- and redox-related genes, including marked upregulation of *FGF2* (up to ~5 log_2_ fold change) and moderate induction of *NOS2*, supporting a state of functional endothelial activation consistent with SEI rather than irreversible injury.

In contrast, methanolic and acetonic extracts induced concentration- and time-dependent cytotoxicity, with IC_50_ values decreasing from 1.55 to 0.91 µg/µL and from 8.94 to 3.82 µg/µL, respectively, between 24 and 48 h. These effects were associated with significant activation of pro-apoptotic signaling (*BAX* up to ~4 log_2_ fold change, *p* < 0.05), downregulation of *P53*, and pronounced cytomorphological features of cell damage. Hexane extracts produced the most severe endothelial alterations, characterized by extensive structural disruption and strong induction of *NOS2* (up to ~5.6 log_2_ fold change, *p* < 0.05), consistent with oxidative and nitrosative stress–mediated endothelial injury.

Importantly, these responses occurred at concentration ranges commonly employed in phytochemical research, indicating that endothelial toxicity and SEI may be underestimated in studies that rely solely on viability-based endpoints. Overall, this work highlights the necessity of solvent-dependent assessment and supports the integration of functional, molecular, and computational approaches to more accurately evaluate the vascular safety and translational potential of *C. cyminum* extracts. Future studies should prioritize compound-level identification, mechanistic validation, and in vivo vascular models to establish safe and effective applications of *C. cyminum*-derived preparations.

## Figures and Tables

**Table 1:** Chemical developers used for qualitative characterization in the preliminary phytochemical analysis (PPA).

Developer	Metabolite Family or Functional Groups to be Identified	Display
Ceric ammoniacal sulfate 1%	Alcohols	Reddish spots on a yellow background
2,4-Dinitrophenylhydrazine	Carbonyl compounds: Aldehydes and ketones	Aldehydes and ketones appear as yellow, orange or red stains
Hager’s reagent	Alkaloids	Red or orange spots
Van-Urk’s reagent	Alkaloids	Alkaloids will appear as blue spots on the plates
Erlich’s reagent	Alkaloids	Pink or reddish spots
Wagner’s reagent	Alkaloids	Spots of red and orange coloration
Potassium permanganate	Alkaloids	Red or orange spots on a yellow background
Ninhydrin	Amino acids	Blue, violet or pink spots
Antrone	Sugars and their derivatives	Carbohydrates appear as yellow, green or blue spots
Sodium nitroprusside-sodium hydroxide	Esters	Red and violet spots
Erlich’s reagent	Indoles and its derivatives	Blue, violet, green and red spots
Reduced methylene blue	Quinones	Blue spots
Diphenylamine-zinc chloride	Chlorinated organic compounds and nucleic acids	Green spots

**Table 2:** Results of the preliminary phytochemical analysis of the hexane, acetonic, methanolic and aqueous extracts of *C. cyminum*.

Developer	Extract			
	Hexane	Acetonic	Methanolic	Aqueous
Ceric ammoniacal sulfate 1%	+++	+++	−	−
2,4-Dinitrophenylhydrazine	+++	+++	++	+
Hager’s reagent	−	−	++	+
Van-Urk’s reagent	+	+	+	+
Erlich’s reagent	−	−	−	−
Wagner’s reagent	+	+	−	−
Potassium permanganate	+++	+++	++	+
Ninhydrin	−	−	−	−
Antrone	−	−	−	−
Sodium nitroprusside-sodium hydroxide	−	−	−	−
Erlich’s reagent	−	−	+	−
Reduced methylene blue	++	+	+	−
Diphenylamine-zinc chloride	−	−	−	−

Qualitative result: positive indicated as +, (increases with the intensity of the signal displayed). Negative −.

**Table 3:** Summary and interpretation of endothelial responses to *Cuminum cyminum* extracts observed in the study.

Extract	Representative Compounds (Present/Enriched)	Key PASS-Predicted Activities (Pa ≥ 0.7)	Gene Expression Pattern (HMEC-1)	Integrated Endothelial Consequence (In Vitro)
Aqueous	Gallic acid, Caffeic acid, Ferulic acid, Quercetin	Membrane integrity agonist; TP53 expression enhancer; JAK2 expression inhibitor; Vasoprotector	↑ *FGF2* (≈5 log_2_); moderate ↑ *NOS2*; no marked ↑ *BAX*; relatively stable *TP53*	Sublethal endothelial adaptation: preserved viability and migration, mild cytoplasmic and membrane remodeling consistent with adaptive stress response
Methanolic	Cuminaldehyde, *p*-Cymene + phenolic acids/flavonoids	HIF1A expression inhibitor; MMP9 expression inhibitor; Apoptosis-associated signaling; Membrane integrity modulation	↑ *BAX*; ↓ *TP53*; ↑ *NOS2*; limited *FGF2* induction	Pro-apoptotic endothelial stress: reduced viability and migration, nuclear abnormalities (pyknosis, hyperchromasia)
Acetonic	Cuminaldehyde, *p*-Cymene, terpenoids + phenolic acids	HIF1A expression inhibitor; JAK2 expression inhibitor; Membrane integrity alteration	↑ *BAX*; ↓ *TP53*; altered *NOS2*; minimal angiogenic signaling	Cytotoxic stress phenotype: concentration-dependent toxicity, impaired migration, nuclear and membrane damage
Hexane	β-Sitosterol, Stigmasterol, lipophilic terpenes and hydrocarbons	HIF1A expression inhibitor; TP53 modulation; Membrane integrity alteration	Strong ↑ *NOS2* (≈5.6 log_2_); dysregulated cell-cycle genes; minimal *FGF2*	Overt endothelial injury: oxidative/nitrosative stress, extensive morphological damage, cellular debris, atypical mitosis

HMEC-1: human microvascular endothelial cells; Pa: probability of activity predicted by PASS Online; FGF2: fibroblast growth factor 2; NOS2: inducible nitric oxide synthase; BAX: BCL2-associated X protein; TP53: tumor protein p53; HIF1A: hypoxia-inducible factor 1 alpha; MMP9: matrix metalloproteinase 9; JAK2: Janus kinase 2. ↑ indicates upregulation and ↓ indicates downregulation relative to control.

## Data Availability

The data presented in this study are available on request from the corresponding authors.

## Supplementary Materials

The following supporting information can be downloaded at: https://www.mdpi.com/article/10.3390/cimb48030255/s1.
