# Supplementary material for: Hallmarks of Sublethal Endothelial Injury Are Differentially Induced by Cuminum cyminum Extracts with Distinct Phytochemical Profiles
Source: Curr Issues Mol Biol. 2026 Feb 26;48(3):255. doi: 10.3390/cimb48030255 (PMC13024819; doi:10.3390/cimb48030255)
Supplement: Supplementary file 1 [file cimb-48-00255-s001.zip › cimb-4139431-supplementary.pdf]

Supplementary Table S1

Table S1. Literature-based phytochemical composition of Cuminum cyminum extracts according to extraction solvent and analytical method.

Extract	Main compound	Chemical class	Evidence of presence in extract	Identification methods reported	Representative biological activities reported	Key references
**Aqueous**	Gallic acid	Phenolic acid	Repeatedly reported	HPLC, LC–MS	Antioxidant, anti-inflammatory	Mnif & Aifa 2015; Al-Hashemi 2014; Srinivasan 2018
	Caffeic acid	Phenolic acid	Repeatedly reported	HPLC	Antioxidant, endothelial protection	Al-Hashemi 2014; Mnif & Aifa 2015
	p-Coumaric acid	Phenylpropanoid	Frequently reported	HPLC	Antioxidant	Al-Hashemi 2014; Sami 2015
	Ferulic acid	Phenolic acid	Frequently reported	HPLC	Cytoprotective, antioxidant	Mnif & Aifa 2015; Sami 2015
	Ellagic acid	Polyphenol	Quantified (low ppm)	HPLC	Antioxidant, anti-inflammatory	El Tannir et al. 2024
	Rutin	Flavonoid	Quantified (trace)	HPLC	Vasoprotective, antioxidant	El Tannir et al. 2024; Mnif & Aifa 2015
	Quercetin	Flavonoid	Repeatedly reported	HPLC, LC–MS	Anti-inflammatory, antioxidant	Mnif & Aifa 2015; Sami 2015
**Methanolic**	Cuminaldehyde	Aromatic aldehyde	Major constituent	GC–MS	Anti-inflammatory, antimicrobial	Chouhan 2021; Chandrasekaran 2023
	p-Cymene	Monoterpene	Repeatedly reported	GC–MS	Antioxidant, antimicrobial	Chouhan 2021; Rahman 2021
	γ-Terpinene	Monoterpene	Repeatedly reported	GC–MS	Antioxidant	Chouhan 2021; Rahman 2021
	D-Limonene	Monoterpene	Repeatedly reported	GC–MS	Anti-inflammatory	Chouhan 2021; Rahman 2021
	Oleic acid	Fatty acid	Frequently reported	GC–MS	Anti-inflammatory, metabolic modulation	Chandrasekaran 2023
**Acetonic**	Cuminaldehyde	Aromatic aldehyde	Reported / inferred by solvent polarity	GC–MS (comparative studies)	Anti-inflammatory	Dorman 2000; Sami 2015
	Phenylpropanoids (e.g., p-coumaric derivatives)	Phenylpropanoids	Reported in polar–apolar solvents	HPLC, GC–MS	Antioxidant	Mnif & Aifa 2015
**Hexane**	Cuminaldehyde	Aromatic aldehyde	Major constituent	GC–MS	Antimicrobial, anti-inflammatory	Gotmar 2018; Singh 2025
	D-Carvone	Oxygenated monoterpene	Major constituent	GC–MS	Antioxidant, anticancer	Rahman 2021; Chandrasekaran 2023
	Apiol	Phenylpropanoid	Repeatedly reported	GC–MS	Antimicrobial	Rahman 2021
	Squalene	Triterpene	Reported	GC–MS	Cytoprotective	Rahman 2021
	Caryophyllene oxide	Sesquiterpene	Reported	GC–MS	Anti-inflammatory	Singh 2025

HPLC, high-performance liquid chromatography; LC–MS, liquid chromatography–mass spectrometry; GC–MS, gas chromatography–mass spectrometry.
